# Modified Lemaire tenodesis reduces anterior cruciate ligament graft forces during internal tibial torque loading

**DOI:** 10.1186/s40634-022-00484-w

**Published:** 2022-05-18

**Authors:** Raul Mayr, Maximilian Sigloch, Christian Coppola, Romed Hoermann, Alessandra Iltchev, Werner Schmoelz

**Affiliations:** 1 Department for Orthopaedics and Traumatology, Medical University of Innsbruck, Anichstraße 35, 6020 Innsbruck, Austria; 2 Institute for Clinical and Functional Anatomy, Medical University of Innsbruck, Innsbruck, Austria

**Keywords:** Knee ligament reconstruction, Anterior cruciate ligament, Lateral extra-articular tenodesis, Modified Lemaire tenodesis technique, Graft force, Load sharing, Biomechanics

## Abstract

**Purpose:**

The aim of the study was to directly measure graft forces of an anterior cruciate ligament reconstruction (ACLR) and a lateral extra-articular tenodesis (LET) using the modified Lemaire technique in combined anterior cruciate ligament (ACL) deficient and anterolateral rotatory instable knees and to analyse the changes in knee joint motion resulting from combined ACLR + LET.

**Methods:**

On a knee joint test bench, six fresh-frozen cadaveric specimens were tested at 0°, 30°, 60°, and 90° of knee flexion in the following states: 1) intact; 2) with resected ACL; 3) with resected ACL combined with anterolateral rotatory instability; 4) with an isolated ACLR; and 5) with combined ACLR + LET. The specimens were examined under various external loads: 1) unloaded; 2) with an anterior tibial translation force (ATF) of 98 N; 3) with an internal tibial torque (IT) of 5 Nm; and 4) with a combined internal tibial torque of 5 Nm and an anterior tibial translation force of 98 N (IT + ATF). The graft forces of the ACLR and LET were recorded by load cells incorporated into custom devices, which were screwed into the femoral tunnels. Motion of the knee joint was analysed using a 3D camera system.

**Results:**

During IT and IT + ATF, the addition of a LET reduced the ACLR graft forces up to 61% between 0° and 60° of flexion (*P* = 0.028). During IT + ATF, the LET graft forces reached 112 N. ACLR alone did not restore native internal tibial rotation after combined ACL deficiency and anterolateral rotatory instability. Combined ACLR + LET was able to restore native internal tibial rotation values for 0°, 60° and 90° of knee flexion with decreased internal tibial rotation at 30° of flexion.

**Conclusion:**

The study demonstrates that the addition of a LET decreases the forces seen by the ACLR graft and reduces residual rotational laxity after isolated ACLR during internal tibial torque loading. Due to load sharing, a LET could support the ACLR graft and perhaps be the reason for reduced repeat rupture rates seen in clinical studies. Care must be taken not to limit the internal tibial rotation when performing a LET.

## Introduction

Injuries to the anterior cruciate ligament (ACL) are often accompanied by deficiencies in its functional synergetic structure, the anterolateral complex [1–4]. Although isolated ACL deficiencies can be successfully treated with ACL reconstruction (ACLR) [5, 6], combined injuries with anterolateral rotatory instability may require additional anterolateral procedures [7]. This augmentation of the ACLR could be important in patients with high-grade pivot shift on examination, when treating young patients participating in pivoting sports, or in the setting of revision ACLR [8, 9]. Clinical studies have shown a higher rate of residual rotational laxity after isolated ACLR in comparison with combined ACLR and anterolateral procedures in anterolateral rotatory instable knees [10, 11]. They also reported repeat rupture rates up to 10% higher with isolated ACLR. This may be due to protection of the ACLR graft by the anterolateral procedure – e.g., a lateral extra-articular tenodesis (LET) – during pivoting manoeuvres via load sharing.

However, there is still controversy over which indications should be used to recommend the addition of a LET, and whether this procedure lead to non-physiologic motion changes in the knee joint. Biomechanical studies have reported controversial results in relation to knee joint motion after combined ACLR + LET. Lagae et al. [12] and Smith et al. [13] recently reported physiological knee motion with combined ACLR + LET. Contrary to this, Slette et al. [14] reported reduced tibial internal rotation in seven out of eight biomechanical studies in a review article. In addition, the functional interaction between anatomic ACLR techniques and a LET concerning graft forces and load sharing has not yet been sufficiently examined.

The purpose of the present biomechanical study was to measure the graft forces of the ACLR and LET under various external loads after isolated ACLR and combined ACLR + LET. Load sharing mechanisms between the LET and an anatomic ACLR were therefore directly quantified for the first time. Knee joint motion after isolated ACLR and combined ACLR + LET was also analysed. The hypotheses of the study were first, that there is a reduction in the ACLR graft forces due to the addition of a LET, in comparison with an isolated ACLR; and second, that the combined ACLR + LET reduces residual rotational laxity that occurs in anterolateral rotatory instable knees after isolated ACLR.

## Materials and methods

### Preparation of specimens

Six fresh-frozen human knee joints from donors with a median age of 86 (range 70–92; 2 male, 4 female) were used for the biomechanical in vitro study. The bodies were donated to the local anatomical institute by individuals who had provided informed consent before death to the use of their bodies for scientific and educational purposes [15].

Quantitative computed tomography scans (LightSpeed VCT 64; GE Healthcare, Chicago, Illinois, USA) were performed to exclude knee arthritis (Kellgren-Lawrence ≥ 3). The specimens were stored at –20 °C and thawed for 24 h at room temperature before testing. Skin and subcutaneous tissue were removed, leaving the knee capsule and inserting tendons intact. A medial parapatellar arthrotomy was performed and closed after ligament integrity was verified. Only specimens without ligamentous and/or bony degeneration or a history of knee injury were included. Under lateral x-ray visualisation, a mediolateral pin was inserted into the distal femur at the intersection between the distal femur cortex and the Blumensaat line, as described by Stannard et al. [16]. This axis approximates the flexion–extension axis in the knee joint. It was used for standardised embedding and positioning inside the test bench [17]. The femur was cut at a distance of 150 mm and the tibia was cut at a distance of 110 mm from the inserted mediolateral pin. The proximal fibula was cut at the fibular neck and fixed to the proximal tibia using two 3.5-mm screws. Reproducible orientation of the knee joint coordinate system relative to the embedding moulds was therefore achieved and allowed knee joint motion analysis via adhesive marker tracking attached to the embedding moulds.

The quadriceps and hamstring tendons were armed with high-strength sutures for active dynamic cyclic motion. Anterior translational forces were applied to the proximal tibia using a circular ring connected to a mediolateral pin placed 65 mm below the knee flexion line. The femur and tibia were embedded in polymethylmethacrylate (PMMA) cement (Technovit 3040, Kulzer GmbH, Wehrheim, Germany) for fixation inside the test bench. While the specimen was being mounted into the test bench, physiological movement of the tibia during dynamic flexion–extension was verified.

### Surgical technique

#### Insufficiency model

The ACL was resected through the medial arthrotomy, which was sutured after each procedure and before testing. The simulated anterolateral rotatory instability consisted of three parts. First, a longitudinal incision was made in the iliotibial band. Second, a cut was created anterior and parallel to the lateral collateral ligament, from the lateral epicondyle to the joint line, in order to cut the anterolateral ligament and capsule, leaving the meniscus and popliteus tendon intact [18, 19]. Thirdly, the distal Kaplan fibres of the deep iliotibial band at the lateral femoral condyle were cut [20, 21].

#### ACLR

All of the surgical procedures were carried out by the same experienced orthopaedic surgeon (RM). ACLR was performed through the medial arthrotomy using bovine digital extensor tendons [22] folded into a two-stranded graft with a diameter of 9 mm. The tibial free ends were secured with baseball stitches (Ethibond no. 2; Ethicon Inc., Raritan, New Jersey, USA). A full 9-mm femoral tunnel was drilled at the centre of the ACL footprint using an anteromedial drill guide (Arthrex Inc., Naples, Florida, USA) with an offset of 7 mm. A full 9-mm tibial tunnel was drilled into the tibial ACL stump using a tibial drill guide set at an angle of 55°. The tunnel length was 40 mm. The tibial graft end was secured with a 9 × 30 mm polyetheretherketone (PEEK) interference screw (Arthrex Inc.), with additional cortical screw fixation of the free graft ends at the proximal tibia cortex. At the femoral side, the graft end was fixed at 30° of flexion with 80 N [12, 23] to a load cell incorporated into a custom device, which was screwed into the femoral tunnel at the distal lateral femur (Fig. 2b).

#### LET

**Fig. 1:**
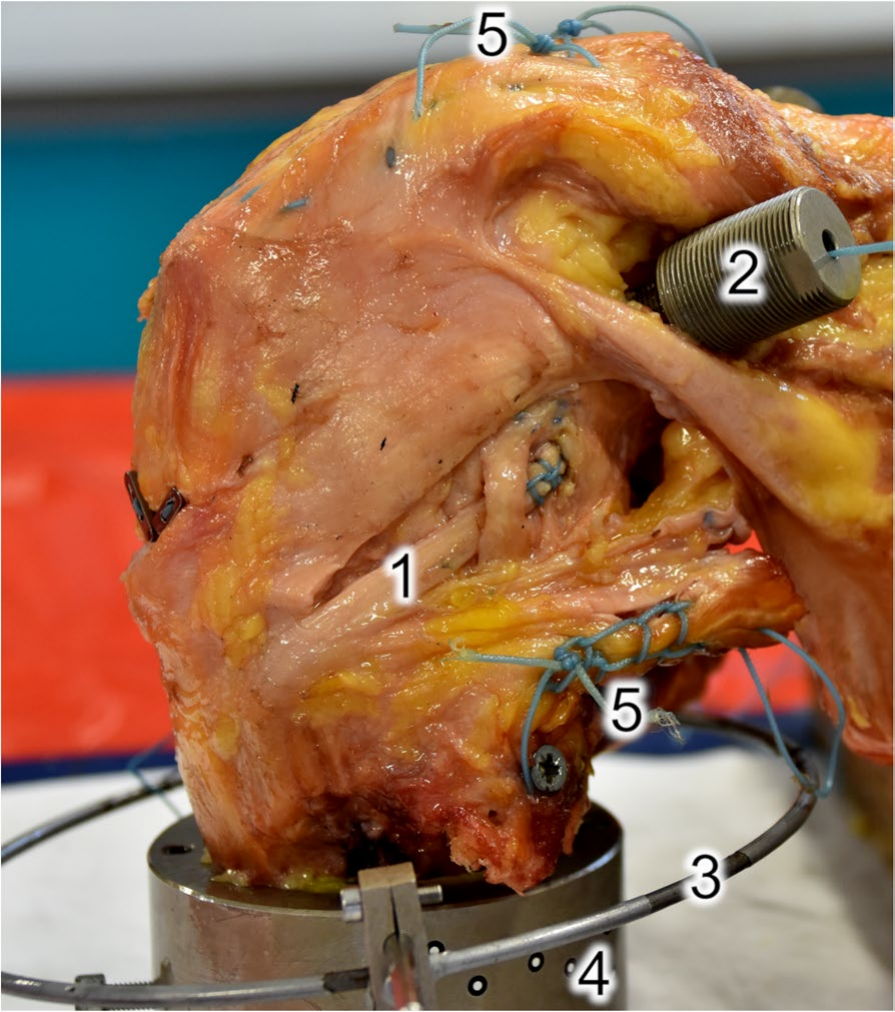
Specimen preparation: 1, lateral extra-articular tenodesis using the modified Lemaire technique with a 10 mm wide strip of the iliotibial band shuttled deep to the lateral collateral ligament and fixed at 60° of flexion with 20 N [23]; 2, cortically fixed part of the custom graft force measuring device (in line with femoral graft tunnels); 3, fixture for applying anterior tibial translation forces, including a tibial ring; 4, embedding mould with optical markers to evaluate knee joint motion; 5, armed muscle tendons (quadriceps and hamstring) for active dynamic preconditioning cycling

A modified Lemaire tenodesis [24] was performed in the study (Fig. 1). A 10 mm wide strip of the central iliotibial band with the fibres oriented centrally toward Gerdy’s tubercle was therefore prepared. The graft was secured with whipstitch sutures (FiberWire #2; Arthrex Inc.). A full femoral tunnel (7 mm in diameter) was positioned 8 mm proximal and 4 mm posterior to the lateral epicondyle in accordance with the Lemaire technique [24, 25]. The graft was shuttled deep to the lateral collateral ligament and fixed at 60° of flexion with 20 N [23] in neutral tibial rotation to another load cell incorporated into a custom device screwed into the LET tunnel at the distal medial femur.

### Testing protocol

The knees were tested in the following states and sequence: 1) intact (removed skin and subcutaneous tissue; closed medial parapatellar arthrotomy); 2) with resected ACL; 3) with resected ACL combined with anterolateral rotatory instability; 4) with isolated ACLR; and 5) with combined ACLR + LET. Each state was investigated without external loading, during application of an anterior tibial translation force (ATF) of 98 N (10 kg), internal tibial torque (IT) of 5 Nm, as well as with a combined internal tibial torque of 5 Nm and anterior tibial translation force of 98 N (IT + ATF). Force and torque values were chosen for comparison reasons to relevant results of recent publications [12, 19, 23, 26]. Investigation of graft forces and amount of anterior translation or internal rotation was carried out at 0°, 30°, 60°, and 90° of flexion.

### Tensioning protocol

The ACLR graft was preconditioned for 10 min under 89 N [27] and fixed at 30° of flexion with 80 N [12, 23]. The LET was fixed at 60° of flexion with 20 N [23]. Before static testing of each state, the respective grafts were re-tensioned and the knee was dynamically cycled 10 times (at 0–75° of flexion) with active muscle forces to allow initial graft preconditioning and relaxation.

### Biomechanical measurements

**Fig. 2:**
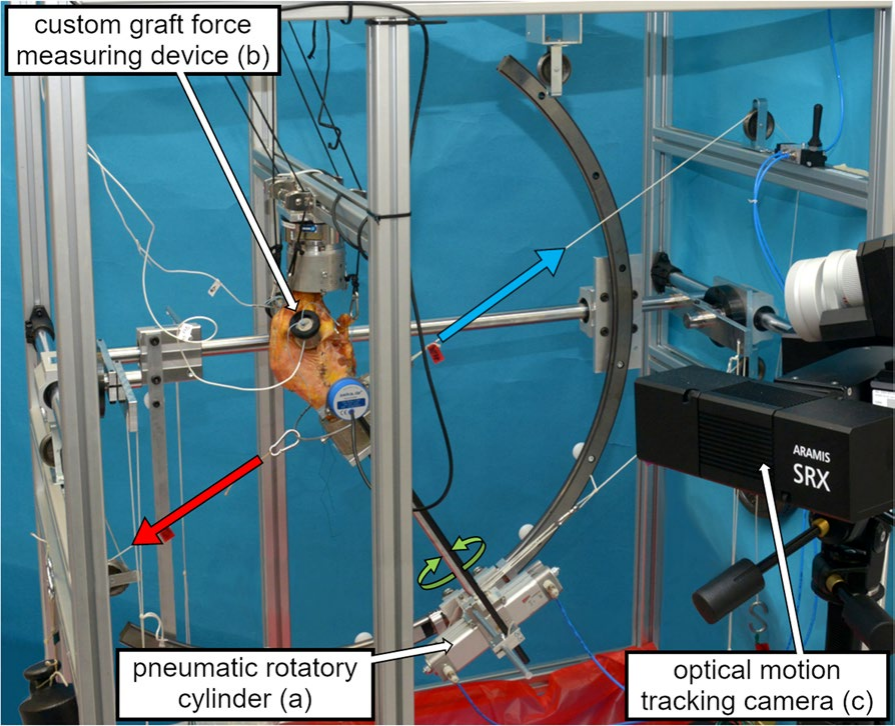
The biomechanical knee joint test bench [17] with a knee specimen fixed at 30° of flexion. Anterior tibial translation force (red arrow) is induced by a pulley-and-weight system. A pnemuatic rotatory cylinder (**a**) is used to apply internal tibial torque (green arrow). The tibial weight is compensated in relation to the flexion angle by another pulley-and-weight system (blue arrow). Graft forces and tibiofemoral joint motion are measured by custom graft force measuring devices (incorparating a load cell) (**b**) and an optical 3D camera system (**c**) respectively

Testing of the different states was conducted on a customised validated test bench with six degrees of freedom (Fig. 2) [17]. Moving the tibia allowed knee flexion to be adjusted to 0°, 30°, 60°, and 90° relative to the fixed femur. Apart from the fixed flexion, the tibia was free to move. The weight of the tibial part was compensated in relation to the flexion angle by dead weights using a pulley system. ATF was induced using another pulley-and-weight system connected to the tibial ring, which could be adjusted to the flexion angle. IT was initiated by a pneumatic rotatory cylinder (Camozzi Automation GmbH, Hall in Tirol, Austria). Graft forces of the ACLR and LET were measured via miniature load cells (Burster Präzisionsmesstechnik GmbH, Gernsbach, Germany) (accuracy: ≤  ± 1%) integrated into the custom devices, which were screwed into the graft tunnels. Graft forces are reported as changes (ΔF) between the unloaded and loaded (AT/IT/IT + ATF) states. Tibiofemoral joint motion was tracked using an optical 3D camera system (ARAMIS SRX, GOM GmbH, Braunschweig, Germany) (accuracy: ≤ 0.1 mm) with adhesive markers attached to the embedding moulds. Anterior translation and internal rotation were calculated relative to the unloaded state of the respective knee condition.

### Data analysis

In combination with a PICAS measuring amplifier (Peekel Instruments GmbH, Bochum, Germany), LabVIEW 11.0 (National Instruments, Austin, Texas, USA) was used to record graft forces. Motion data were analysed using ARAMIS Professional 2018 (GOM GmbH, Braunschweig, Germany). Statistical analyses were conducted in RStudio (RStudio Inc., Boston, Massachusetts, USA) with R version 4.0.1. The significance level was set to 0.05. Because of the limited sample size (*n* = 6), a non-normal distribution was assumed. A related-samples two-sided Wilcoxon signed rank test was used for every flexion angle in order to test for significant differences between graft force data for the isolated ACLR and combined ACLR + LET. Friedman’s two-way analysis of variance (ANOVA) by ranks with Conover’s post hoc test and Holm-Bonferroni correction for multiple comparisons [28] was used to compare the results of the knee joint motion analysis between the five tested states, with the flexion angle as an independent variable. Median values are reported in the results. Bootstrapping (*n* = 1000) was used to calculate 95% confidence intervals.

## Results

### Graft forces of the ACLR and LET

**Fig. 3:**
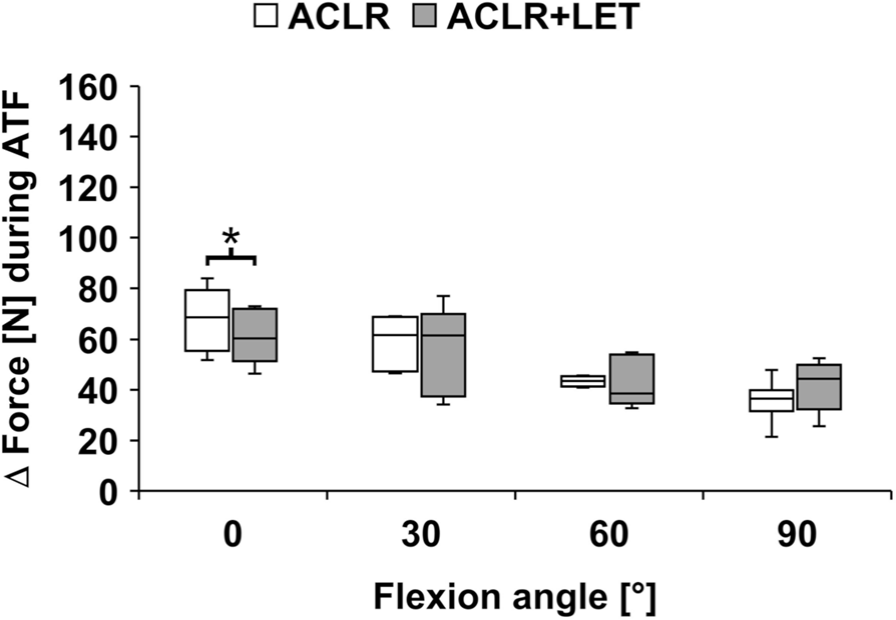
Graft forces in the anterior cruciate ligament reconstruction (ACLR) during the application of anterior tibial translation force (ATF) for the isolated ACLR and the ACLR with addition of a lateral extra-articular tenodesis (ACLR + LET). The forces indicated in the graph are relative to the unloaded state (Δ force). Significant differences between the two procedures are marked with an asterisk (*)

During ATF, the ACLR graft forces in combined ACLR + LET were significantly lower compared to the isolated ACLR at 0° of flexion (*P* = 0.028). There were no significant differences between the two states (isolated ACLR vs. combined ACLR + LET) for any of the other flexion angle tested (*P* > 0.25) (Fig. 3).

**Fig. 4:**
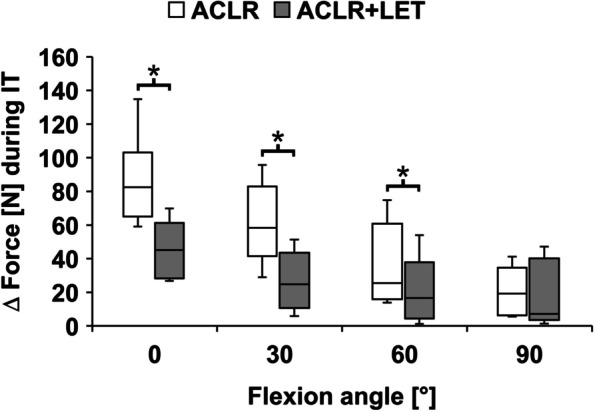
Graft forces in the anterior cruciate ligament reconstruction (ACLR) during the application of internal tibial torque (IT) for the isolated ACLR and the ACLR with addition of a lateral extra-articular tenodesis (ACLR + LET). The forces indicated in the graph are relative to the unloaded state (Δ force). Significant differences between the two procedures are marked with an asterisk (*)

**Fig. 5:**
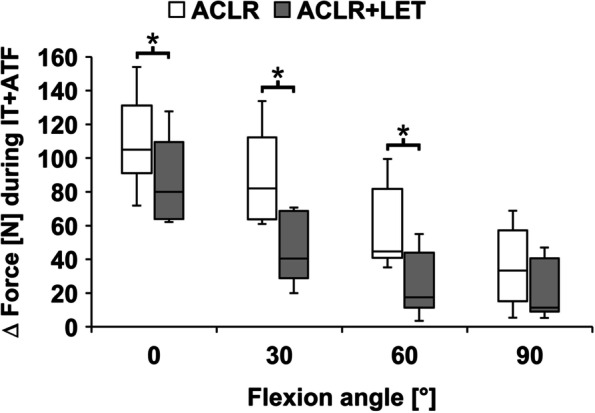
Graft forces in the anterior cruciate ligament reconstruction (ACLR) during the application of combined internal tibial torque and anterior tibial translation force (IT + ATF) for the isolated ACLR and the ACLR with addition of a lateral extra-articular tenodesis (ACLR + LET). The forces indicated in the graph are relative to the unloaded state (Δ force). Significant differences between the two procedures are marked with an asterisk (*)

During IT and IT + ATF, the ACLR graft forces in combined ACLR + LET were significantly lower compared to the isolated ACLR at 0°, 30°, and 60° (*P* = 0.028) (Figs. 4 and 5). With increasing flexion angle, the overall ACLR graft forces as well as the absolute differences between the two states (isolated ACLR vs. combined ACLR + LET) decreased. For 90° flexion, the ACLR graft forces were comparable, with no significant differences (IT: *P* = 0.463, IT + ATF: *P* = 0.116). The average force reduction over all tested flexion angles was 49% during IT (0°: 45%; 30°: 61%; 60°: 50%; 90°: 39%), 45% during IT + ATF (0°: 17%; 30°: 52%; 60°: 60%; 90°: 52%) and 7% during ATF (0°: 12%; 30°: 4%; 60°: 11%; 90°: 0%).

**Fig. 6:**
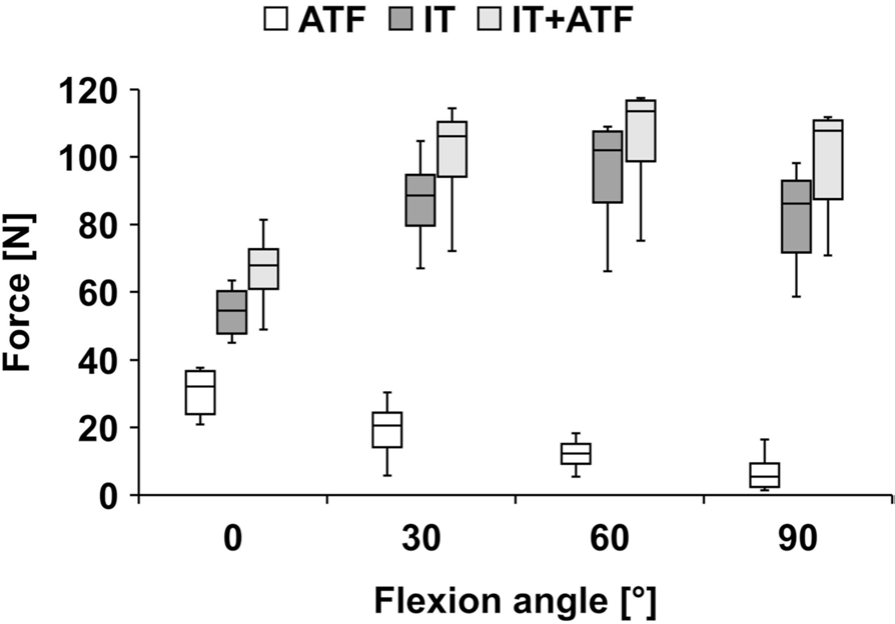
Absolute graft forces in the lateral extra-articular tenodesis during the application of anterior tibial translation force (ATF), internal tibial torque (IT), and combined loading (IT + ATF)

During ATF, the absolute LET graft forces decreased with flexion angle. During IT and IT + ATF, the absolute LET graft forces increased up to 60° (Fig. 6). During ATF, the LET graft forces were greatest at full extension, with a median of 32 N (95% CI, 23 to 37 N). During IT and IT + ATF, the LET graft forces were greatest at 60° flexion, with a median of 102 N (95% CI, 80 to 106 N) and 112 N (95% CI, 91 to 117 N), respectively.

### Knee joint motion

**Fig. 7:**
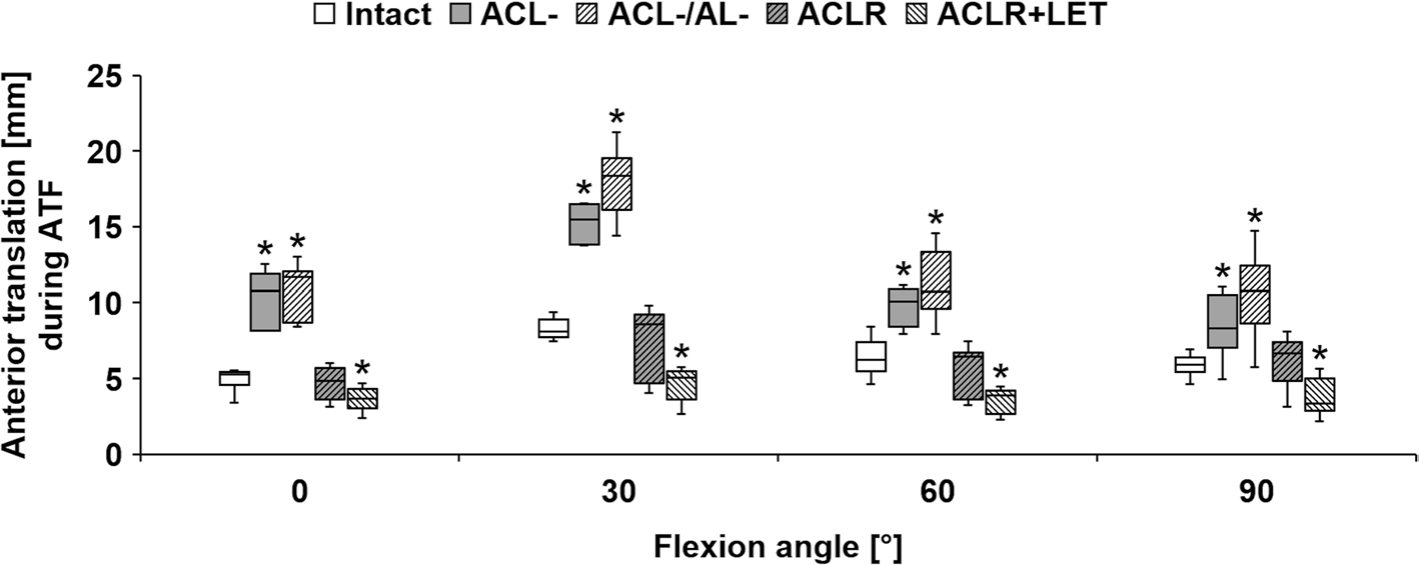
Anterior translation during the application of anterior tibial translation force (ATF), in comparison between the intact state, state with resected anterior cruciate ligament (ACL-), state with resected ACL combined with anterolateral rotatory instability (ACL-/AL-), state with ACL reconstruction (ACLR), and state with ACLR and addition of a lateral extra-articular tenodesis (ACLR + LET) for different angles of knee flexion. Significant differences from the intact state are marked with an asterisk (*) for each flexion angle

**Fig. 8:**
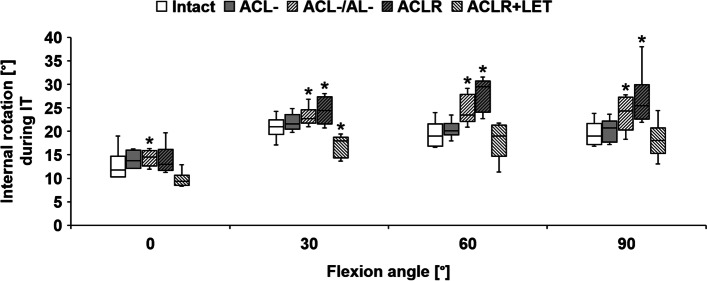
Tibial rotation during the application of internal tibial torque (IT), in comparison between the intact state, state with resected anterior cruciate ligament (ACL-), state with resected ACL combined with anterolateral rotatory instability (ACL-/AL-), state with ACL reconstruction (ACLR), and state with ACLR and addition of a lateral extra-articular tenodesis (ACLR + LET) for different angles of knee flexion. Significant differences from the intact state are marked with an asterisk (*) for each flexion angle

**Fig. 9:**
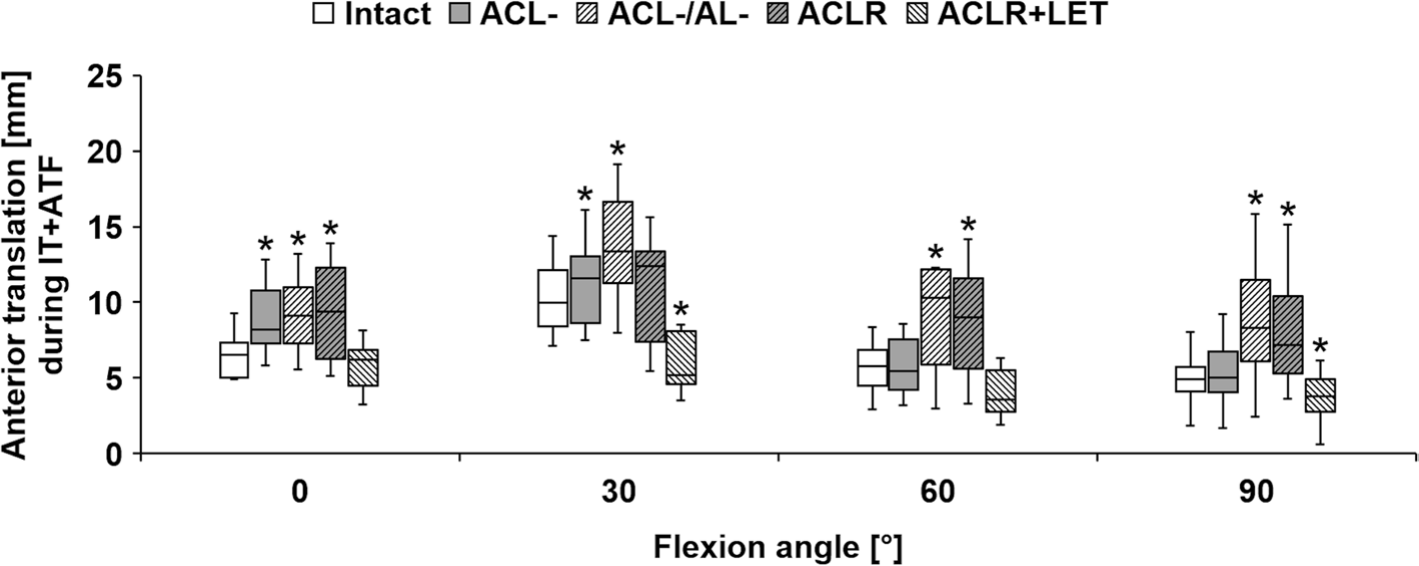
Anterior translation during the application of combined internal tibial torque and anterior tibial translation force (IT + ATF), in comparison between the intact state, state with resected anterior cruciate ligament (ACL-), state with resected ACL combined with anterolateral rotatory instability (ACL-/AL-), state with ACL reconstruction (ACLR), and state with ACLR and addition of a lateral extra-articular tenodesis (ACLR + LET) for different angles of knee flexion. Significant differences from the intact state are marked with an asterisk (*) for each flexion angle

Cutting of the ACL significantly increased anterior translation during ATF for every tested flexion angle (*P* < 0.001) (Fig. 7). In contrast, this did not significantly increase the internal rotation during IT (0°: *P* = 0.11, 30°: *P* = 0.32, 60°: *P* = 0.92, 90°: *P* = 0.41) (Fig. 8). Cutting of the ACL significantly increased anterior translation during IT + ATF at 0° (*P* = 0.003) and 30° (*P* = 0.01) of flexion (Fig. 9).

Further sectioning of the anterolateral structures (combined deficiency) led to a significant increase compared to the isolated ACL deficiency in both anterior translation during ATF (0°: *P* = 0.003; 30°: *P* = 0.003; 90°: *P* = 0.002) and internal rotation during IT (30°: *P* = 0.02; 60°: *P* = 0.003; 90°: *P* = 0.002). For 60° of flexion there was no significant additional increase in anterior translation during ATF (*P* = 0.08). For 0° of flexion, internal rotation during IT did not differ significantly from the isolated ACL deficient state (*P* = 0.72), but combined with the non-significant increase of the ACL deficiency did differ significantly from the native state (*P* = 0.02). The combined deficiency significantly increased anterior translation during IT + ATF compared to the native state for all tested flexion angles (0°/30°/90°: *P* < 0.001, 60°: *P* = 0.01).

The isolated ACLR was able to nearly approximate the native anterior translation during ATF for every tested flexion angle without significant differences (0°: *P* = 1.00, 30°: *P* = 1.00, 60°: *P* = 0.28, 90°: *P* = 0.22). The isolated ACLR was not able to restore native internal rotation during IT for any tested flexion angle (*P* < 0.001), except for 0° flexion (*P* = 0.41). A non-significant increase in median internal rotation during IT between the combined deficient and isolated ACLR state was seen at 30° to 90°. In order to directly measure graft forces, small soft tissue incisions had to be done to securely fixate the custom graft force measuring device, which enhanced the lateral insufficiency. The isolated ACLR was able to restore the native anterior translation during IT + ATF at 30° (*P* = 0.07) but not at 0° (*P* < 0.001), 60° (*P* = 0.02), or 90° (*P* = 0.01).

Addition of the LET (combined ACLR + LET) and re-tensioning of the ACLR graft in between states significantly decreased the anterior translation during ATF in comparison with the isolated ACLR as well as the native state for all tested flexion angles (*P* < 0.001). The combined ACLR + LET led to a significant reduction in internal rotation during IT in comparison with the isolated ACLR, restoring native motion for 0° (*P* = 0.07), 60° (*P* = 0.92), and 90° (*P* = 0.15). At 30° of flexion, the combined ACLR + LET resulted in significantly decreased internal rotation in comparison with the native state (*P* = 0.01). The combined ACLR + LET reduced anterior translation during IT + ATF, resulting in non-significant differences from the native state at 0° (*P* = 0.62) and 60° (*P* = 0.15). For 30° (*P* = 0.007) and 90° (*P* = 0.02), combined ACLR + LET significantly differed from the native state during IT + ATF due to decreased anterior translation.

## Discussion

The most important finding of the present study was, that the addition of a LET fixed at 60° of flexion with 20 N using the modified Lemaire technique was found to reduce ACLR graft forces significantly for loadings of the knee joint that involved IT (retaining the first hypothesis). When an ATF was applied, the addition of a LET played a minor role and did not have a major influence on ACLR graft forces. The LET decreased residual rotational laxity after isolated ACLR, approximating native internal rotation under the tested conditions (retaining the second hypothesis). Unphysiological time-zero limitation of internal rotation was observed at 30° of flexion.

### Graft forces of the ACLR and LET

The present study has directly measured forces in the LET (modified Lemaire technique) and ACLR grafts during different loading conditions. During loadings involving IT, measured LET graft forces reached high values of up to 112 N and ACLR graft forces were decreased from 0° to 60° of knee flexion when comparing the isolated ACLR to the combined ACLR + LET. This effect of the LET on the ACLR graft could support the ACLR due to load sharing and was directly quantified for the first time.

Engebretsen et al. [29] reported on ACLR graft force reduction due to the addition of an anterolateral procedure as early as 1990. In contrast to the present study, addition of the anterolateral procedure significantly reduced ACLR graft forces by a mean of 43% during ATF (90 N). The authors used an over-the-top femoral ACLR, and the anterolateral graft was fixed at 30° of flexion. The data are therefore not directly comparable with those presented in the present study, and this might explain the differences.

Noyes et al. [30] also directly measured the forces of an ACLR graft in combination with anterolateral ligament reconstruction (ALLR). The ALLR was able to significantly reduce ACLR graft forces under 5 Nm of internal torque for all knee flexion angles tested, up to 76% (mean of 67%). As in the results of the present study, the ACLR graft forces decreased with increasing flexion angles during internal torque.

Another way of determining graft forces is to use a robotic system and the principle of superposition combined with serial sectioning [31], as described by various research groups. Using this technique, Novaretti et al. [32] and Marom et al. [26] recently investigated ACL forces in the context of anterolateral procedures. In contrast to the present results, both studies observed a significant reduction of ACLR (respectively ACL) forces when combined with an anterolateral procedure for flexion angles over 60° in response to an ATF (mean of 54%). For angles under 60°, no significant differences were evident. The discrepancy may be explained by methodological differences. The graft was pre-tensioned at 44 N (in comparison with 20 N) in the study by Marom et al. [26], and the ATF was 134 N (in comparison with 98 N) in the study of Novaretti et al. [32]. Both of these factors might increase the influence of the anterolateral procedure, since it was either higher tensioned per se or the ACL had to resist a greater external load. In addition, Novaretti et al. [32] used an ALLR fixed at 30° of flexion and left the ACL intact. In the present study, an ATF of 98 N was chosen in order to allow comparison with biomechanical studies investigating ACLR and LET using the modified Lemaire technique [12, 19, 26, 33]. Fixing the LET at 60° of flexion with a preload of 20 N in neutral tibial rotation was reported to approximate physiological knee joint motion with nearly isometric graft behaviour using the modified Lemaire technique [23, 25]. Furthermore, this tensioning protocol is used in our clinical practice. It must be mentioned that results from biomechanical studies may differ with different tensioning parameters.

The study by Marom et al. [26] is the only biomechanical study investigating graft forces that has also examined combined ACLR + LET using the modified Lemaire technique. The authors’ results are largely consistent with the present ones. Particularly for loading with IT, the LET was able to reduce ACLR graft forces significantly. At increasing flexion angles, they also reported an increase in LET graft forces and a decrease in ACLR graft forces. As in the present study, the ACLR mainly counteracted anterior tibial translation forces, and the LET played an inferior role. In contrast, the ACLR was only of secondary importance restricting internal tibial rotation. All of the mentioned studies investigating internal tibial rotation concluded that the addition of an anterolateral procedure is able to reduce ACLR graft forces during IT loadings due to load sharing [26, 30, 32]. This is consistent with the present data and could be the reason for reduced repeat rupture rates seen in clinical studies as the LET might possibly protect the ACLR graft in special cases like young patients participating in pivoting sports [34] or in the setting of revision ACLR [35].

### Knee joint motion

The isolated ACLR was able to restore native knee joint motion during application of an ATF, but left a residual rotational laxity in combined ACL deficient and anterolateral rotatory instable knees. This finding is consistent with biomechanical and clinical studies reporting that isolated ACLR is not able to restore native knee joint motion in all cases [10, 19, 36, 37]. Fixation of the graft force measuring device slightly enhanced the lateral insufficiency, possibly underestimating the effect of the isolated ACLR during IT. Due to the test setup, this limitation could not be eliminated but tried to keep as small as possible to not significantly influence the results. Addition of a LET (combined ACLR + LET) fixed with 20 N of tension at 60° of flexion appears to provide an internal tibial rotation comparable with those of the native knee at most knee flexion angles. In the present study, decreased internal tibial rotation was only observed at 30° of flexion. Various other studies have also investigated reduced knee joint motion after the addition of an anterolateral procedure [26, 33, 37, 38]. The results of the present study might lead to the conclusion that even lower pre-tensioning of the LET might be reasonable in the future. However, the long-term effects of reduced tibial rotation on tibiofemoral or meniscal loading are as yet unknown.

In the present study, combined ACLR + LET showed anterior tibial translation below native values during ATF and IT + ATF. This might be mainly because of the re-tensioning protocol used for the ACLR graft. Preconditioning and tensioning values for the grafts were selected to be as close as possible to clinical practice, in order to increase the validity of the in vitro testing. Due to irreversible viscoelastic effects in vitro (stress relaxation and slack increase), as well as the stress history dependency of biological tissue, the temporally separate graft force measurements in the two states (isolated ACLR and combined ACLR + LET) are not directly comparable [39–42]. It was therefore decided to re-tension the ACLR before investigating the combined treatment (ACLR + LET), in order to avoid inherent overestimation of the effect of an LET addition on ACLR graft forces and evaluate the change between the unloaded and loaded states (ΔF) instead of absolute values. Re-tensioning also affected tibial anterior translation as the isolated ACLR alone was able to decrease anterior translation significantly and re-tensioning of the ACLR graft enhanced the decrease. This was considered acceptable, as tibial anterior translation was not the focus of the present study.

### Limitations

As this was an in vitro biomechanical study, only the time-zero condition of the postoperative state – without healing, graft integration, or any other in vivo processes – could be investigated. Furthermore, only the knee joint itself, without the foot and ankle or skin and subcutaneous tissue, was examined. There was no muscle induced stabilisation or iliotibial band loading during the static investigations which resulted in high tibial translational and rotational values. As ligament-based stability strongly relates to muscle loaded stability and is a reliable predictor for functional stability [43], it was chosen to not load any muscle in order to limit influencing factors and analyse the true ligamentous behaviour. In addition, care needs to be taken in transferring the results to all anterolateral procedures, as they vary in the position of the femoral graft or take a different treatment approach in the case of an ALLR. Moreover, the anterolateral rotatory instability model with complete disruption of the iliotibial band used in the present study represents a profound injury to the anterolateral complex and could lead to higher rotational laxity as typically seen clinically. Results are therefore particularly important in special cases when patients with high-grade pivot shift laxities on examination are treated. Fixation of the custom graft force measuring device had the tendency to enhance the lateral insufficiency of the anterolateral rotatory instability model. The small sample size is also a limitation of the study. In the present study, bovine tendons were used because they have mechanical properties comparable to human hamstring tendons not influencing the biomechanical measurements [22]. They were also easily accessible and provided more homogeneous graft sizes [44]. Another limitation is the donor age for the specimens tested, as the donors do not represent the typical patient receiving an anterolateral procedure. However, a quantitative computed tomography scan of possible specimens was conducted, and arthritic specimens were not selected. Pure anterior translation of the tibia is likely different from the conducted anterior translation of the lateral plateau. Re-tensioning the ACLR graft had an influence on the graft forces as well as joint motion, as discussed above.

## Conclusions

The addition of a LET using the modified Lemaire technique was found to provide a significant reduction in ACLR graft forces in comparison with the isolated ACLR for internal tibial torque loading. With anterolateral rotatory instability, isolated ACLR did not restore internal tibial rotation. After combined ACLR + LET, internal tibial rotation was comparable to that of the native knee, with decreased internal rotation at 30° of flexion. LET treatment in combination with an ACLR could therefore be helpful in postoperative protection of the ACLR during rotational loadings.

## Data Availability

Data are available upon reasonable request.

## Abbreviations

ACL: Anterior cruciate ligament
ACLR: Anterior cruciate ligament reconstruction
ALLR: Anterolateral ligament reconstruction
ATF: Anterior tibial translation force
IT: Internal tibial torque
LET: Lateral extra-articular tenodesis
